# Idiopathic Upper Tracheal Stenosis Presenting With Progressive Stridor Managed by Endoscopic Balloon Dilatation: A Case Report

**DOI:** 10.7759/cureus.106019

**Published:** 2026-03-28

**Authors:** Krishna Mohan Kaimal

**Affiliations:** 1 Otolaryngology - Head and Neck Surgery, Royal Blackburn Teaching Hospital, Blackburn, GBR

**Keywords:** balloon dilatation, endoscopic airway surgery, idiopathic subglottic stenosis, stridor, tracheal stenosis

## Abstract

Idiopathic subglottic and upper tracheal stenosis is an uncommon cause of progressive airway obstruction, predominantly affecting middle-aged women. We report the case of a female patient who presented with progressive stridor at rest over 12 months, without antecedent airway instrumentation or systemic disease. Flexible nasoendoscopy demonstrated irregular subglottic mucosa with airway narrowing, and computed tomography confirmed a 1.5 cm segment of circumferential mucosal thickening resulting in significant luminal compromise. Comprehensive autoimmune screening was negative, supporting an idiopathic aetiology. The patient underwent endoscopic balloon dilatation with adjunctive mucosal trimming, resulting in immediate symptomatic improvement. This case highlights the importance of thorough diagnostic evaluation and minimally invasive management of idiopathic upper tracheal stenosis.

## Introduction

Idiopathic subglottic and upper tracheal stenosis is a rare fibroinflammatory condition characterised by progressive narrowing of the airway in the absence of identifiable aetiological factors such as trauma, prolonged intubation, infection, or systemic inflammatory disease. It predominantly affects middle-aged women and often presents with insidious onset dyspnoea and stridor, frequently leading to delayed diagnosis [1].

Exclusion of secondary causes, particularly granulomatosis with polyangiitis and connective tissue disease, is essential prior to establishing an idiopathic diagnosis [1].

Endoscopic balloon dilatation is an established minimally invasive treatment option for selected patients with subglottic and tracheal stenosis [2]. Systematic reviews have evaluated endoscopic treatment modalities, including balloon dilatation and adjunctive techniques, for idiopathic subglottic stenosis [3].

We present a case of grade III [4] idiopathic upper tracheal stenosis successfully managed with endoscopic balloon dilatation, illustrating diagnostic challenges and therapeutic considerations.

## Case presentation

A 40-year-old female patient was referred to the otolaryngology clinic with a 12-month history of progressively worsening exertional dyspnoea and stridor at rest. There was no history of previous neck surgery, prolonged intubation, airway trauma, or systemic inflammatory disease.

**Figure 1:**
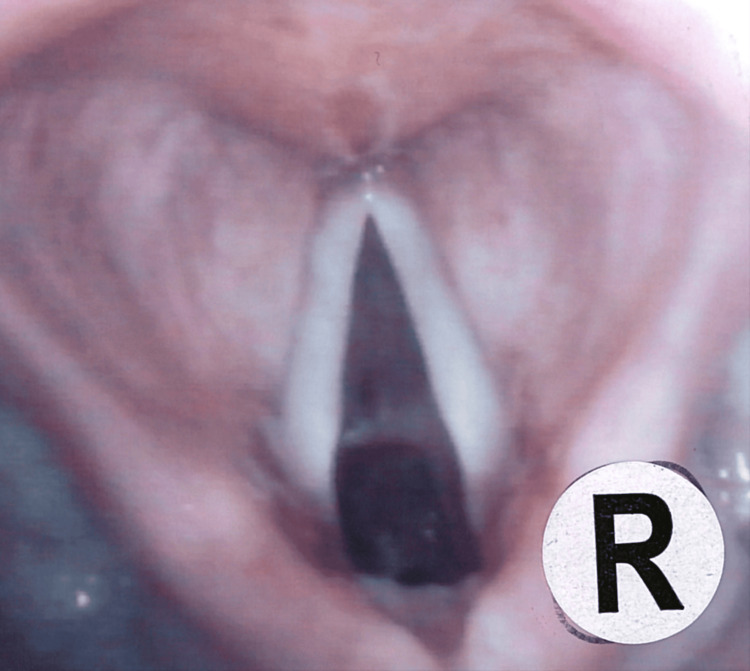
Endoscopic view of the subglottic stenosis

Clinical examination demonstrated mild expiratory wheeze and audible stridor at rest. Flexible nasoendoscopy revealed irregular mucosa involving the subglottic region and anterior tracheal wall with significant airway narrowing (Figure 1). 

The distal extent of stenosis was difficult to visualise due to endoscopic angulation.

**Figure 2:**
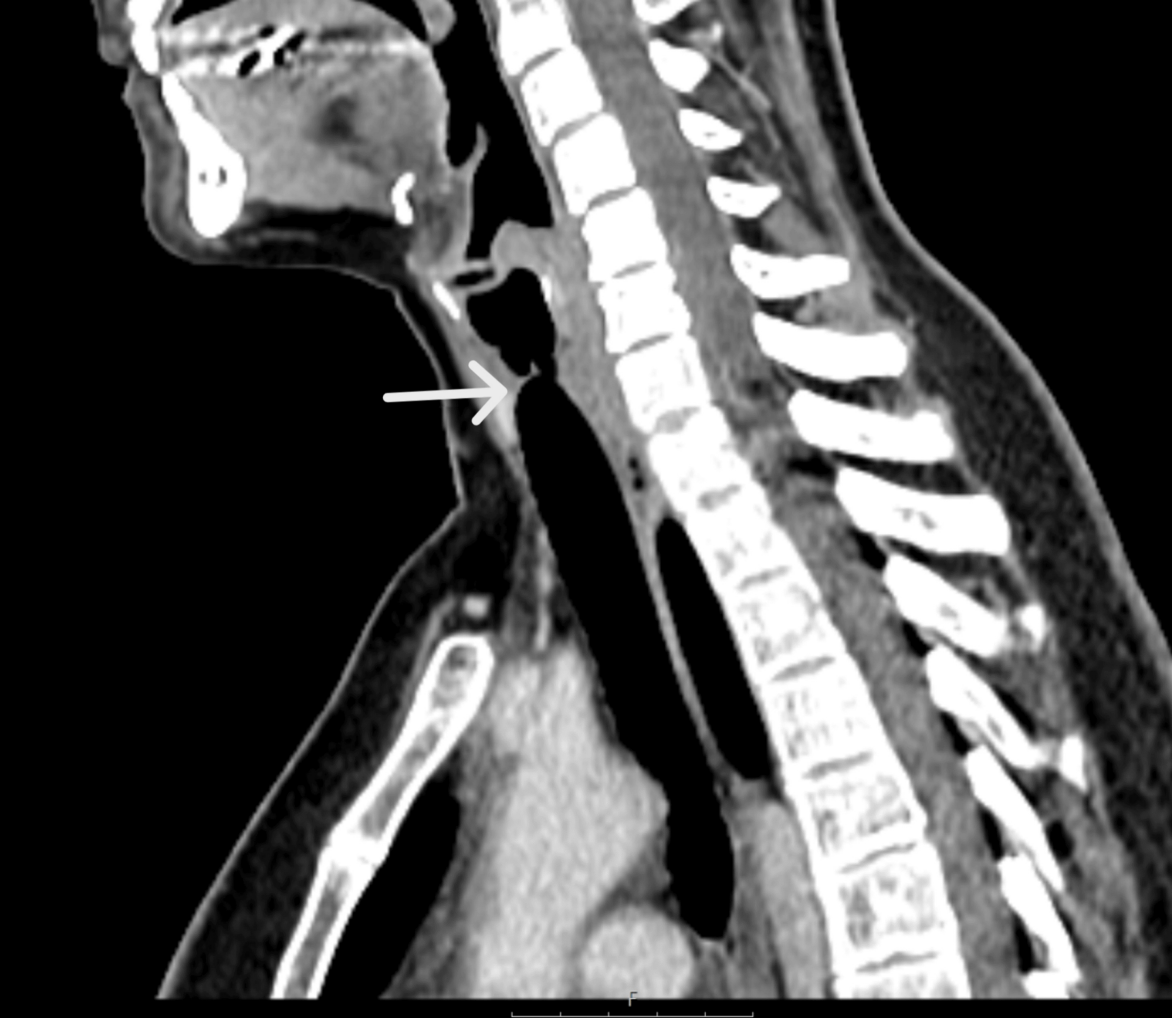
Sagittal CT showing short-segment stenosis

Contrast-enhanced computed tomography of the neck demonstrated circumferential mucosal thickening extending from the inferior margin of the cricoid cartilage into the proximal trachea, measuring approximately 1.5 cm in cranio-caudal length. The narrowest luminal diameter measured approximately 5 mm (Figure 2). 

**Figure 3:**
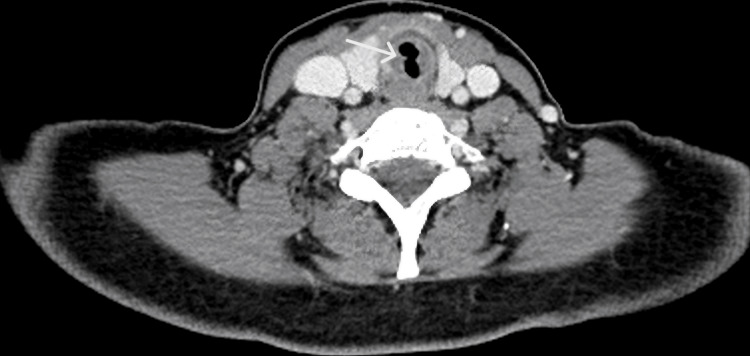
Axial CT showing upper tracheal stenosis at the narrowest point

**Figure 4:**
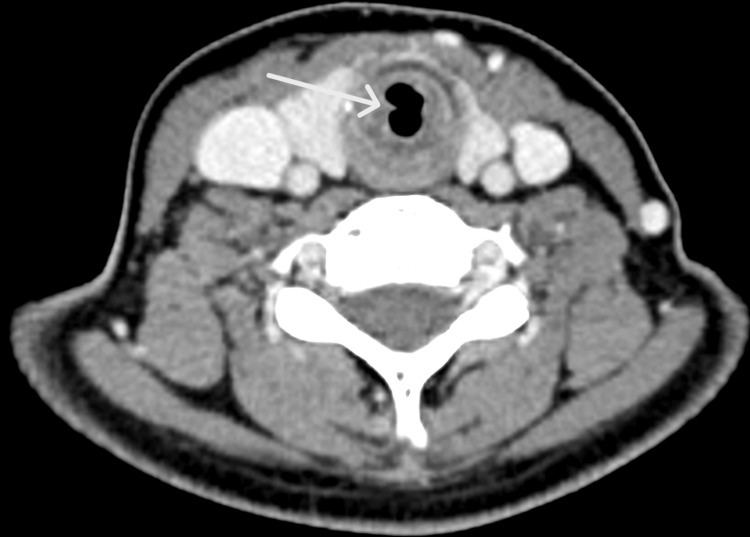
Additional axial CT slice demonstrating the extent of the stenosis

Adjacent axial slices further demonstrated the short-segment nature of the stenosis without distal tracheal involvement (Figures 3, 4). 

Autoimmune screening was performed to exclude inflammatory and vasculitic aetiologies. Anti-neutrophil cytoplasmic antibodies, including proteinase-3 and myeloperoxidase antibodies, were within normal limits. Connective tissue disease screening, including antinuclear antibodies and extended extractable nuclear antigen panel, was negative. These findings supported a diagnosis of idiopathic upper tracheal stenosis.

Based on clinical, radiological, and serological findings, the stenosis was graded as Cotton-Myer grade III [4]. The patient was consented for endoscopic management and underwent urgent balloon dilatation. The patient was maintained under total intravenous anaesthesia (TIVA) with spontaneous ventilation and no neuromuscular blockade. Oxygenation was provided using high-flow nasal oxygen via an Airvo^TM^ 2 system (Fisher & Paykel Healthcare, East Tāmaki, Auckland, New Zealand), while the stenotic segment was exposed with a Lindholm laryngoscope, allowing continuous oxygen delivery during endoscopic balloon dilatation.

Under general anaesthesia, a Lindholm laryngoscope was used to expose the stenotic segment. Visualisation was achieved using a 0-degree Hopkins rod telescope. Serial balloon dilatation was performed using 12 mm, 13.5 mm, and 15 mm balloons at pressures of 3, 4.5, and 8 atmospheres respectively. Adjunctive mucosal trimming was performed using a laryngeal coblator to remove redundant tissue. A satisfactory airway lumen was achieved without intraoperative complications. The patient did not receive systemic corticosteroid therapy prior to the procedure or during the immediate postoperative period.

Postoperatively, the patient experienced immediate symptomatic improvement and was discharged the same day. Follow-up was arranged at six weeks, with advice to attend emergency services if acute airway compromise developed. 

## Discussion

This case highlights the importance of maintaining a high index of suspicion for upper airway pathology in patients presenting with progressive stridor in the absence of prior intubation or airway trauma. Idiopathic subglottic and upper tracheal stenosis is a recognised but uncommon entity, predominantly affecting middle-aged women and frequently presenting with delayed diagnosis due to gradual symptom progression [1].

A comprehensive autoimmune workup was undertaken in this patient, given the radiological appearance of mucosal thickening and the need to exclude inflammatory causes such as granulomatosis with polyangiitis. Negative anti-neutrophil cytoplasmic antibodies (ANCA) and connective tissue disease screening supported a diagnosis of idiopathic stenosis after secondary causes were excluded [1]. Establishing an idiopathic aetiology was important in guiding management strategy and counselling regarding prognosis.

Congenital airway anomalies such as tracheal web may present with short-segment airway narrowing; however, these typically manifest earlier in life and appear radiologically as thin membranous diaphragms rather than circumferential mucosal thickening [5]. The adult onset of symptoms and imaging findings in this case support an acquired idiopathic stenosis rather than a congenital lesion.

Endoscopic balloon dilatation is an accepted minimally invasive treatment option for selected patients with subglottic and tracheal stenosis and is supported by national procedural guidance [2]. Given the short-segment nature of the stenosis in this case, an endoscopic approach was favoured over open reconstruction.

Systematic reviews have demonstrated symptomatic improvement following endoscopic management of idiopathic subglottic stenosis, although recurrence remains a recognised feature and repeat intervention may be required [3]. Accordingly, long-term endoscopic surveillance was planned for this patient in view of the recognised potential for recurrence even after successful initial dilatation. These considerations were discussed with the patient during counselling.

Radiological assessment confirmed a short-segment stenosis of approximately 1.5 cm without distal tracheal involvement. Established classification systems for adult laryngotracheal stenosis assist in guiding management decisions, with high-grade symptomatic stenosis typically requiring intervention [5]. In this case, grade III luminal compromise with stridor at rest mandated active treatment.

Serial graded balloon dilatation allowed controlled expansion of the airway lumen. Adjunctive coblation was used to contour irregular mucosa and optimise airway patency. Immediate postoperative improvement confirmed restoration of functional airway calibre. Structured follow-up with interval endoscopic surveillance was arranged given the recognised relapsing nature of idiopathic disease [1,3].

This case reinforces the importance of systematic exclusion of secondary causes, careful radiological evaluation of stenosis length, and tailored selection of minimally invasive techniques in appropriately selected patients.

## Conclusions

Idiopathic upper tracheal stenosis is an uncommon but clinically significant cause of progressive airway obstruction. Accurate diagnosis requires careful exclusion of secondary inflammatory and traumatic causes. In this case, comprehensive serological evaluation and imaging allowed confident diagnosis of short-segment idiopathic stenosis.

Endoscopic balloon dilatation provided effective immediate airway restoration with minimal morbidity. The success of this approach was influenced by the limited cranio-caudal extent of disease and the absence of distal involvement. Long-term follow-up remains essential due to the recognised risk of recurrence. This case underscores the importance of systematic diagnostic evaluation and individualised management planning in adult subglottic stenosis.
